# Age at menarche and general and abdominal obesity in older Brazilian women: ELSI-Brazil

**DOI:** 10.11606/s1518-8787.2026060007243

**Published:** 2026-07-06

**Authors:** Nair Tavares Milhem Ygnatios, Juliana Vaz de Melo Mambrini, Juliana Lustosa Torres, Núbia Carelli Pereira de Avelar, Ana Lúcia Danielewicz, Rosana Carolina Souza Duarte de Matos, Luciana de Souza Braga, Maria Fernanda Lima-Costa, Bruno de Souza Moreira

**Affiliations:** I Centro Universitário Santa Rita. Conselheiro Lafaiete, MG, Brasil; II Núcleo de Estudos em Saúde Pública e Envelhecimento. Belo Horizonte, MG, Brasil; III Fundação Oswaldo Cruz. Instituto René Rachou. Belo Horizonte, MG, Brasil; IV Universidade Federal de Minas Gerais. Faculdade de Medicina. Departamento de Medicina Preventiva e Social. Belo Horizonte, MG, Brasil; V Universidade Federal de Santa Catarina. Departamento de Fisioterapia. Araranguá, SC, Brasil

**Keywords:** Menarche, Waist Circumference, Body Mass Index, Obesity, Women's Health

## Abstract

**OBJECTIVE:**

To examine the association between age at menarche and general and/or abdominal obesity in Brazilian women aged ≥ 50 years.

**METHODS:**

Cross-sectional study conducted with data from the second wave of the Brazilian Longitudinal Study of Aging (*Estudo Longitudinal da Saúde dos Idosos Brasileiros*(ELSI-Brazil, 2019–2021), in a nationally representative sample of community-dwelling individuals aged ≥ 50 years. Self-reported age at menarche was categorized as ≤ 12 years, 13–15 years, and ≥ 16 years. General obesity was defined as a body mass index of ≥ 30 kg/m^2^, and abdominal obesity as a waist circumference of ≥ 88 cm. The variables were combined into "general or abdominal obesity" and "general and abdominal obesity". Multinomial logistic regression adjusted for sociodemographic and lifestyle characteristics was used.

**RESULTS:**

Among the 4,229 participants, 41.1% (95% confidence interval —95%CI 37.9–44.3) reported menarche at ≤ 12 years and 9.3% (95%CI 8.0–10.9) at ≥ 16 years. The prevalence of general or abdominal obesity was 36.6% (95%CI 33.7–39.5), while the prevalence of general and abdominal obesity was 35.0% (95%CI 32.5–37.7). After adjustments, women with age at menarche of ≤ 12 and 13–15 years had, respectively, 84% (odds ratio — OR = 1.84; 95%CI 1.14–2.98) and 64% (OR = 1.64; 95%CI 1.04–2.57) greater odds of general and abdominal obesity compared with those with menarche at ≥ 16 years. There was no significant association between age at menarche and general or abdominal obesity.

**CONCLUSION:**

Menarche before age 16 is associated with a higher odds of general and abdominal obesity combined, but not in isolation, suggesting its potential as a relevant marker for the formulation of public policies aimed at preventing obesity in girls from school age onwards.

## INTRODUCTION

Menarche, defined as the first menstrual period, is a key physiological event in females that occurs during puberty, marking the onset of the reproductive cycle and the transition from childhood to adulthood^1^. The global average age at menarche is 12 years^1^. However, it often ranges from ten to 16 years, influenced by both genetic and environmental factors that differ between populations^2^. The criteria used to define early or late menarche vary considerably across studies, which may hinder comparability and consistency of findings. It is worth noting that these divergences may reflect specific contextual characteristics of different populations, including socioeconomic and environmental changes, which can, over time, affect age at menarche.

The literature has demonstrated a positive association between early menarche and increased body fat in adulthood. For example, a study of women aged 45–54 years from the Baltimore, Maryland, found that each year of increase in age at menarche was associated with a 31 and 34% reduction in the odds of obesity (body mass index [BMI] 30 kg/m^2^–34.9 kg/m^2^) and severe obesity (BMI ≥ 35 kg/m^2^), respectively^3^. Similarly, the risk of obesity decreased with increasing age at menarche in women aged 49–83 years who participated in the Swedish Mammography cohort^4^. Additional evidence from studies conducted in Chinese (30–79 years)^5^, US (≥ 40 years)^6^, and Indian (20–45 years)^7^ women confirm that early menarche was associated with increased body adiposity. However, the underlying mechanisms are not fully understood. One possible explanation for this relationship is the increased androgen concentrations resulting from earlier menarche, which may contribute to the development of obesity at older ages^8^.

However, most previous studies have assessed overall obesity (excessive accumulation of body fat)^9^, which is based on the calculation of BMI, with limited data on other important anthropometric measures of regional body fat distribution, such as waist circumference (WC), which reflects the accumulation of abdominal fat (abdominal obesity)^10^. Although these anthropometric measures are correlated, they may indicate differences in risk factors for cardiovascular disease, especially between the sexes^11^. It is essential to highlight that aging leads to changes in body composition, such as loss of lean mass and an increase in abdominal fat, which may be more relevant than BMI in determining the health risk associated with obesity in later life^12^. It is known that measures of abdominal obesity, such as WC, are better predictors of cardiovascular disease and its risk factors^13^ in older adults compared to BMI.

Furthermore, in Brazil, there is little evidence on the relationship between reproductive history and adiposity in older women. Studies found in the literature are limited to local samples, which do not accurately represent the diversity of the Brazilian population. In the South of this country, early menarche (≤ 11 years) was a strong predictor of general and abdominal obesity in women aged 40–65 years^14^. A more recent study, conducted with representative and independent samples of women aged 20–60 years living in the urban area of the municipality of São Leopoldo, Rio Grande do Sul, evaluated in 2003 and 2015, identified that early menarche (8–11 years) was associated with a higher prevalence of abdominal obesity in both assessments^15^.

Additionally, the World Health Organization highlights the importance of including this topic in global health agendas^16^. Although age at menarche is not a modifiable factor, its link to lifetime obesity is very important for public health. Evidence shows that early menarche can be influenced by environmental exposures, socioeconomic conditions, and genetic factors that affect metabolic and cardiovascular risks in adulthood^17^. Understanding this connection can help identify more vulnerable groups, allowing for targeted interventions on modifiable risk factors such as dietary habits, physical activity, and healthcare access. Research in this field is needed to reveal disparities in menstrual health among different populations and to support the development of public policies aimed at preventing obesity in girls from a young age. Therefore, the objective of this study was to examine the association between age at menarche and general and/or abdominal obesity in Brazilian women aged 50 years and over, using data from a comprehensive national survey. Our hypothesis is that early menarche would be associated with general and abdominal obesity in middle-aged and older women, regardless of sociodemographic and lifestyle characteristics.

## METHODS

### Participants and Study Design

For this cross-sectional study, data from the second wave of the Brazilian Longitudinal Study of Aging (*Estudo Longitudinal da Saúde dos Idosos Brasileiros* — ELSI-Brazil), conducted between 2019 and 2021, were used. This research was conducted in a nationally representative sample of non-institutionalized adults aged 50 years and over, residing in 70 municipalities across the five Brazilian geographic macroregions. ELSI-Brazil is a longitudinal study launched in 2015 to investigate the dynamics of aging in the Brazilian population and its determinants. Sampling was performed in strata based on the population size of the municipalities. Between 2019 and 2021, the final study sample comprised 9,949 participants, of whom 5,898 (54.4%) were female. In this study, we excluded 592 women due to missing data on age at menarche and 276 participants whose answers to questions about women's health were provided with the assistance of a proxy. Additionally, 801 women were excluded due to missing information on anthropometric measurements, resulting in a total of 4,229 women included in the analyses. Further details on the methodology of the first and second waves of ELSI-Brazil can be found in other publications^18,19^ and on the research homepage^a^.

### Age at Menarche

Age at menarche was assessed by the following question: "At what age did you get your first period?". For this analysis, the response options were categorized into three groups: "≤ 12 years", "13 to 15 years", and "≥ 16 years". The literature presents great variability regarding the categorization of the age at menarche, with no globally defined cut-off points. In the present study, therefore, the categorization of the age at menarche was guided by two main criteria, considering the categories previously established in the ELSI-Brazil questionnaire and the empirical distribution of data in the analyzed sample.

### Anthropometric Measurements

Anthropometric measurements were obtained using standardized protocols. In this analysis, we used the average of two measurements. Weight was measured using a portable digital scale (Seca^®^, model 813); height was measured using a portable vertical stadiometer (Nutri-Vida^®^); and WC was assessed using a tape measure (Seca^®^, model 201). Participants who were bedridden or used wheelchairs did not have their anthropometric measurements taken^20^. General obesity was assessed using the BMI, calculated by dividing body weight (in kilograms) by height (in meters) squared (kg/m^2^), and classified according to the World Health Organization cutoff point of BMI ≥ 30 kg/m^2^.^9^ Abdominal obesity was defined as a WC of ≥ 88 cm^9^. For the analyses, a variable was created combining BMI and WC, categorized as "no general and abdominal obesity", "general or abdominal obesity", and "general and abdominal obesity". The adoption of this approach is consistent with the most recent consensus recommendations on obesity^11^, which aim to identify a clinically significant obesity profile.

### Adjustment Variables

Study adjustment variables included: age group in years (50–59; 60–69; 70–79; ≥ 80); education in completed years of study (0–4; 5–8; ≥ 9); monthly household income *per capita* in tertiles (from poorest to richest); alcohol consumption (never/less than once a month; once or more a month); smoking assessed using two questions: "In your lifetime, have you smoked at least 100 cigarettes?" and "Do you currently smoke?"; participants were categorized as: never smoked (those who had not smoked 100 cigarettes throughout their lives), former smokers (those who stated that they had smoked at least 100 cigarettes throughout their lives and who did not currently smoke), and current smokers (those who stated that they had smoked at least 100 cigarettes throughout their lives and who currently smoked) — this classification is widely used in population studies and recommended by international guidelines^18^; and physical activity, measured according to the time spent walking and in moderate and vigorous activities^21^, assessed using the short version of the International Physical Activity Questionnaire, which was previously translated and validated for the Brazilian population^22^ (sedentary; active, considering participants who performed 150 minutes or more of physical activity in the last week, according to World Health Organization recommendations^23^).

### Statistical Analysis

First, the prevalence of sociodemographic and lifestyle characteristics was estimated for the total sample and according to age at menarche. Then, we performed a descriptive analysis of age at menarche and sociodemographic and lifestyle characteristics according to general and/or abdominal obesity. Differences were verified using Pearson's chi-square test with Rao-Scott correction, considering a significance level of 5%. The same test was used to compare the study's variables among excluded and included participants. The association between age at menarche and general and/or abdominal obesity was assessed using a multinomial logistic regression analysis, in which crude and mutually adjusted odds ratios (OR) for sociodemographic and lifestyle characteristics were estimated, along with their respective 95% confidence intervals (95%CI). The possible collinearity between education and monthly household income *per capita* was assessed using the variance inflation factor (VIF), and no relevant collinearity was identified (VIF = 1.25). In this analysis, the group "no general and abdominal obesity" was used as the reference category. Finally, we plotted on a graph the predicted probability of general and/or abdominal obesity by age at menarche, estimated using multinomial logistic regression and adjusted for sociodemographic and lifestyle characteristics. The analyses were performed using Stata/SE software (StataCorp, College Station, United States), version 17.0, with the survey (svy) command to account for the complexity of the sampling design and the individual weights of ELSI-Brazil participants. This study was reported in accordance with the Strengthening the Reporting of Observational Studies in Epidemiology (STROBE) guidelines for cross-sectional studies.

### Ethical Aspects

ELSI-Brazil was approved by the Ethics Committee of the Fundação Oswaldo Cruz — Minas Gerais, and the process is registered on the Plataforma Brasil (Certificate of Presentation for Ethical Appreciation — CAAE: 34649814.3.0000.5091). Participants signed an informed consent form for each research procedure.

## RESULTS

Of the 4,229 women included in this study, 41.1% (95%CI 37.9–44.3) reported an age at menarche of ≤ 12 years, 49.6% (95%CI 47.0–52.2) between 13 and 15 years, and 9.3% (95%CI 8.0–10.9) ≥ 16 years. The prevalence of general or abdominal obesity was 36.6% (95%CI 33.7–39.5), while the prevalence of general and abdominal obesity was 35.0% (95%CI 32.5–37.7).

Table 1 presents a description of sociodemographic and lifestyle characteristics for the total sample, as well as by age at menarche. Regarding sociodemographic characteristics, 49.3% of participants were between 50 and 59 years old, and 49.6% had 0–4 years of education. Regarding lifestyle variables, most participants reported consuming alcohol never/less than once a month (91.3%), never smoked (71.2%), and were physically active (50.1%). The relative distributions of the variables age group and smoking were significantly different between the age at menarche groups: older women (≥ 80 years) and former smokers had a higher frequency of late menarche (≥ 16 years). Additionally, to assess potential biases arising from excluding participants with missing data, we conducted a comparative analysis of the women included and those excluded from the analytical sample. These comparisons showed that the excluded participants were older (≥ 80 years: 14.4% *versus* 5.2%; p-value < 0.001), had lower education levels (0–4 years of education: 56.3% *versus* 49.6%; p-value = 0.0152), and a higher frequency of physical inactivity (sedentary: 63.2% *versus* 49.9%; p-value < 0.001) than the ones included. Furthermore, a higher proportion of women without general and abdominal obesity was observed among the excluded participants (33.9% *versus* 28.4% among included women; p-value = 0.0047). On the other hand, the distribution of age at menarche was similar between the excluded and included participants (≤ 12 years: 43.5% *versus* 41.1%; p-value = 0.6113).

**Table 1:** Description of sociodemographic and lifestyle characteristics for the total sample and according to age at menarche among Brazilian women aged 50 years and over. Brazilian Longitudinal Study of Aging (ELSI-Brazil, 2019–2021).

Variables		Total (n = 4,229)^a^	Age at menarche			p-value^b^
			≤ 12 years (n = 1,658)^a^	13 to 15 years (n = 2,142)^a^	≥ 16 years (n = 429)^a^	
Age group (years), %						
	50–59	49.3	52.9	48.1	39.2	**< 0.001**
	60–69	30.1	27.4	31.4	35.2	
	70–79	15.4	14.5	15.5	19.0	
	≥ 80	5.2	5.2	5.0	6.6	
Education (years), %						
	0–4	49.6	50.2	48.0	55.6	0.363
	5–8	21.2	20.5	22.4	17.6	
	≥ 9	29.2	29.3	29.6	26.8	
Monthly household income *per capita*, %						
	1^st^ tertile	35.6	35.5	35.5	36.7	0.500
	2^nd^ tertile	34.4	34.5	33.5	38.5	
	3^rd^ tertile	30.0	30.0	31.0	24.8	
Alcohol consumption, %						
	Never/less than once a month	91.3	90.1	92.5	90.1	0.235
	Once or more a month	8.7	9.9	7.5	9.9	
Smoking, %						
	Never smoked	71.2	70.4	72.6	67.4	**0.020**
	Former smokers	17.4	16.7	17.0	22.8	
	Actual smokers	11.4	12.9	10.4	9.8	
Physical activity, %						
	Sedentary	49.9	50.8	50.9	40.6	0.058
	Active	50.1	49.2	49.1	59.4	

Notes: all estimates considered the complex sample design and individual weights.

In bold: p-value < 0.05.

a Number of interviewees, not including corrections according to sampling parameters and study design.

b p-value estimated using Pearson's χ^2^ test with Rao-Scott correction.

Table 2 displays the prevalence of age at menarche and sociodemographic and lifestyle characteristics according to general and/or abdominal obesity. There was a statistically significant difference between the groups in the variables age group, smoking, and physical activity.

**Table 2:** Prevalence of age at menarche and sociodemographic and lifestyle characteristics according to general and/or abdominal obesity among Brazilian women aged 50 years and over. Brazilian Longitudinal Study of Aging (ELSI-Brazil, 2019–2021).

Variables		No general and abdominal obesity (n = 1,176)^c^	General^a^ or abdominal^b^ obesity (n = 1,621)^c^	General^a^ and abdominal^b^ obesity (n = 1,432)^c^	p-value^d^
Age at menarche (years), %					
	≤ 12	27.2	35.1	37.7	0.059
	13 to 15	28.1	37.6	34.3	
	≥ 16	35.2	37.4	27.4	
Age group (years), %					
	50–59	30.5	34.3	35.2	**0.015**
	60–69	26.7	35.3	38.0	
	70–79	25.4	43.4	31.2	
	≥ 80	27.7	45.3	27.0	
Education (years), %					
	0–4	26.6	38.7	34.7	0.216
	5–8	30.1	33.3	36.6	
	≥ 9	30.3	35.3	34.4	
Monthly household income *per capita*, %					
	1^st^ tertile	28.1	36.6	35.3	0.568
	2^nd^ tertile	27.5	35.6	36.9	
	3^rd^ tertile	29.3	38.1	32.6	
Alcohol consumption, %					
	Never/less than once a month	28.2	36.5	35.3	0.690
	Once or more a month	30.4	37.5	32.1	
Smoking, %					
	Never smoked	29.2	35.6	35.2	**< 0.001**
	Former smokers	19.4	39.6	41.0	
	Actual smokers	37.0	38.2	24.8	
Physical activity, %					
	Sedentary	24.8	37.7	37.5	**0.008**
	Active	31.4	35.2	33.4	

Notes: all estimates considered the complex sample design and individual weights.

In bold: p-value < 0.05.

a Body mass index ≥ 30 kg/m^2^.

b Waist circumference ≥ 88 cm.

The results of the crude and adjusted associations between age at menarche and general and/or abdominal obesity are presented in Table 3. After adjusting for sociodemographic and lifestyle characteristics, age at menarche was positively associated with general and abdominal obesity, with a dose-response gradient. It was observed that women who reported age at menarche up to 12 years and 13 to 15 years had, respectively, 84% (OR = 1.84; 95%CI 1.14–2.98) and 64% (OR = 1.64; 95%CI 1.04–2.57) greater odds of general and abdominal obesity compared to those with an age at menarche of ≥ 16 years.

**Table 3:** Association between age at menarche and general and/or abdominal obesity among Brazilian women aged 50 years and over. Brazilian Longitudinal Study of Aging (ELSI-Brazil, 2019–2021).

Age at menarche	General^a^ or abdominal^b^ obesity		General^a^ and abdominal^b^ obesity	
	Crude model OR (95%CI)	Adjusted model^c^ OR (95%CI)	Crude model OR (95%CI)	Adjusted model^c^ OR (95%CI)
≤ 12 years (*versus* ≥ 16 years)	1.21 (0.87–1.70)	1.25 (0.88–1.78)	**1.77 (1.12–2.80)**	**1.84 (1.14–2.98)**
13 to 15 years (*versus* ≥ 16 years)	1.26 (0.93–1.70)	1.32 (0.97–1.79)	**1.57 (1.00–2.45)**	**1.64 (1.04–2.57)**

OR: odds ratio, 95%CI: 95% confidence interval, estimated by the multinomial logistic regression model.

Reference category: no general and abdominal obesity. All estimates considered the complex sample design and individual weights. Total n (unweighted) of the adjusted model = 3,858.

In bold: p-value<0.05.

a Body mass index ≥ 30 kg/m^2^.

b Waist circumference ≥ 88 cm.

c Adjusted for age group, education, monthly household income *per capita*, alcohol consumption, smoking, and physical activity.

Figure illustrates the predicted probabilities estimated based on the adjusted model, showing higher estimates of general and abdominal obesity for women with an age at menarche of ≤ 12 years (38.2%) compared to those with an age at menarche of ≥ 16 years (27.6%).

**Figure:**
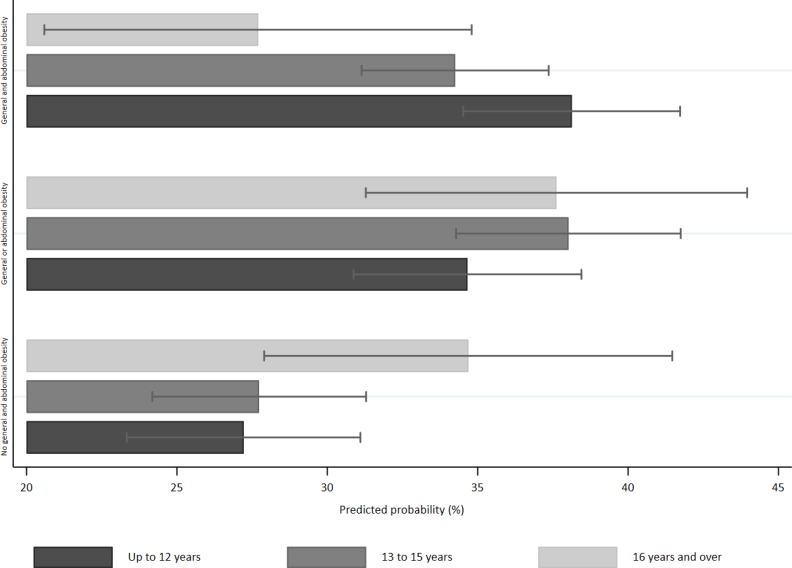
Predicted probability* of general^a^ and/or abdominal^b^ obesity according to age at menarche among Brazilian women aged 50 years and over. Brazilian Longitudinal Study of Aging (ELSI-Brazil, 2019–2021).

* Estimated by the multinomial logistic regression model adjusted for age group, education, monthly household income per capita, alcohol consumption, smoking, and physical activity. The bars represent the 95% confidence interval.

^a^ Body mass index ≥ 30 kg/m^2^.

^b^ Waist circumference ≥ 88 cm.

## DISCUSSION

This study showed that the age at menarche among older Brazilian women was independently associated with general and abdominal obesity. Specifically, participants with menarche up to 15 years of age were more likely to have both types of obesity (general and abdominal obesity) compared to those whose menarche occurred at 16 years and over, regardless of sociodemographic and lifestyle factors at the time of the interview. However, this association was not observed when participants had only one type of obesity. The high percentage of women reporting an age at menarche of ≤ 12 years in this study (41.1%) deserves attention, as this may indicate a cohort effect, especially in upper-middle-income countries like Brazil, where participants experienced periods of transition with improved living conditions and health.

‘Our results are consistent with those of other studies. In Brazil, regional studies carried out in the south of the country found that early menarche is an important factor associated with general and abdominal obesity in middle-aged and older women (40–65 years)^14^ and is related to a higher prevalence of abdominal obesity in women aged 20–60 years^15^. Evidence from other population contexts, involving US (45–54 years)^3^ (≥ 40 years)^6^, Swedish (49–83 years)^4^, Chinese (30–79 years)^5^, and Indian (20–45 years)^7^ women, reinforce the association between early menarche and greater body adiposity in adulthood.

Menarche, an indicator of sexual maturation, signals the beginning of significant physical and psychological changes resulting from increased sex steroids and growth hormone. This process is triggered by the secretion of gonadotropin-releasing hormone by the hypothalamus, which activates the hypothalamic-pituitary-gonadal axis, stimulating the production of sex steroids^24^. In this sense, our results suggest that age at menarche may serve as a proxy for endogenous exposure to sex hormones, and that a more prolonged exposure to estrogen due to early menarche could contribute to greater body fat accumulation in older women^25,26^.

A Mendelian randomization study indicates that, in addition to hormonal influence, early menarche may contribute to increased BMI through psychological factors^27^. Girls who experience menarche at an early age face the challenge of quickly adapting to bodily changes and social expectations of their peers. This situation can lead to high levels of stress, mental health problems, body dissatisfaction, and eating disorders^28,29^. Possible explanations for this relationship include emotional eating behaviors and weight gain secondary to antidepressant use^27^. Furthermore, there is evidence that symptoms of eating disorders can persist and extend beyond puberty, with lasting impacts^29^.

The intense changes that occur at the onset of adolescence can also lead to behaviors that are detrimental to health. Early menarche is often associated with binge eating^29^. Unhealthy habits developed during adolescence can harm lifestyles later in life^30^, potentially contributing to the development of obesity. It is essential to acknowledge that unhealthy health behaviors are often reflective of a family's socioeconomic status and perceived social support, which have both direct and indirect effects on age at menarche^30^, underscoring the complexity of this relationship. Some authors have also noted that shared genetic factors may influence both age at menarche and predisposition to obesity^31^. Additionally, a population-based survey conducted in Norway found a positive association between early menarche (≤ 12 years) and chronic generalized musculoskeletal complaints in adulthood^32^. It is widely recognized that musculoskeletal pain is linked to several conditions in older adults, including disability, low physical activity level, reduced mobility, depression, and poor sleep quality^33^, which, in turn, may coexist with obesity. Our results, which show an association between early menarche and greater body adiposity in middle and older age, may be related to unhealthy behaviors established during adolescence, since the literature^29,34,35^ suggests that girls who experience early menarche tend to engage in binge eating and participate less in physical activities — both of which contribute to weight gain over the life course. These mechanisms help to explain, at least in part, the findings observed in the current study.

We found no significant association between age at menarche and general or abdominal obesity. This result suggests that specific factors associated with the combination of these two types of obesity may reflect a more particular profile of body fat accumulation, with greater relevance in the relationship with age at menarche. This distinction is crucial, considering that abdominal fat, assessed by WC, is strongly associated with metabolic and cardiovascular risks^13,36^, serving as a more sensitive marker at advanced ages compared to BMI. This approach is consistent with The Lancet Diabetes & Endocrinology Commission, which highlights the importance of using complementary anthropometric measurements in obesity assessment^11^. On the other hand, the intermediate category "general or abdominal obesity" is varied, grouping women with different patterns of body fat accumulation — those with overall excess weight but no fat buildup in the abdominal area, and those with isolated abdominal fat; this variability may have attenuated the associations in this group.

Thus, our findings reinforce the importance of simultaneously considering both the quantity and distribution of body fat in older women. In this context, future studies are needed to investigate the mechanisms underlying the observed association between age at menarche and the concomitant presence of general and abdominal obesity. Understanding these factors may contribute to the development of more targeted prevention strategies that address modifiable risk factors from the early stages of life.

The strengths of this study include its large sample size and the use of objective measures of body adiposity, which provide greater robustness to the results. In addition, controlling for sociodemographic and lifestyle characteristics in adulthood allowed us to minimize the effect of potential confounding factors. On the other hand, our study has some limitations that should be mentioned. First, the study's cross-sectional design does not allow causal inferences, making it impossible to determine the temporal order between exposure and outcome. Furthermore, the results may be subject to reverse causality, since childhood obesity can precede early menarche. However, the database does not contain information on pre-menarche nutritional status. It is possible that some residual confounding persists due to unmeasured factors, including socioeconomic conditions and childhood nutrition, as well as other contextual determinants. In addition, we opted to conduct the analysis using a complete-case study approach, which may introduce selection bias, especially if participants with missing data were excluded non-randomly, potentially affecting the magnitude of the observed associations. In this context, the comparison between included and excluded participants showed that excluded women were older, had lower education levels, were more often inactive, and differed in nutritional status. However, the distribution of age at menarche was similar between the included and excluded women. Since this is the main exposure variable in the present study, differential selection errors are reduced. Additionally, age at menarche was obtained through self-report, which may be subject to recall problems, particularly among older participants. However, there is evidence of high reproducibility of this information in older women^37^.

The results of this study have practical implications. Age at menarche can serve as a helpful marker in identifying women at greater risk of developing combined obesity throughout their lives. Health professionals should consider age at menarche as a relevant factor for proposing preventive strategies against obesity from the early stages of life. From a public health perspective, incorporating this marker into prevention programs and actions can help in the early identification of girls who are more vulnerable to adverse health outcomes, contributing to the planning of more effective policies to combat obesity from youth to old age.

In conclusion, our results revealed that age at menarche ≤ 15 years was positively associated with the simultaneous presence of general and abdominal obesity among Brazilian women aged ≥ 50 years. On the other hand, no significant association was observed when these obesity conditions were analyzed in isolation. These findings suggest that, in clinical practice, assessing overall and abdominal fat accumulation jointly, using BMI and WC, is essential for a more comprehensive and accurate understanding of the relationship between female reproductive life cycle factors and body fat distribution at older ages. The novel scientific contribution of this study arises from considering key methodological aspects, including the categorization of age at menarche and the combined assessment of general and abdominal obesity. Together, these expand knowledge on the topic and offer valuable insights for public health.

## Data Availability

Data will be made available upon request on the ELSI-Brazil homepage (https://elsi.cpqrr.fiocruz.br/en/home-english/en-data-access/).
